# From Observation to Surgery: A Review of Literature and an Updated Algorithm for Acquired Retinoschisis and Schisis-Detachment

**DOI:** 10.3390/medsci14010159

**Published:** 2026-03-23

**Authors:** Alessandra Scampoli, Tomaso Caporossi

**Affiliations:** 1 Ophthalmology Department, Isola Tiberina Hospital—Gemelli Isola, Via di Ponte Quattro Capi, 39, 00186 Rome, Italy; 2 Ophthalmology Department, Catholic University “Sacro Cuore”, Largo Francesco Vito, 00168 Rome, Italy

**Keywords:** retinoschisis, retinal detachment, scleral buckling, pars plana vitrectomy, ultra-widefield OCT

## Abstract

This review critically synthesizes current evidence regarding the natural history, advanced diagnostic imaging, and therapeutic interventions for acquired retinoschisis and retinoschisis-associated retinal detachment. A systematic search of PubMed and Embase databases was conducted for literature published up to 2026, focusing on comparative outcomes of scleral buckling versus pars plana vitrectomy and novel imaging modalities. The advent of ultra-widefield optical coherence tomography has shifted the diagnostic paradigm, enabling the precise identification of outer layer breaks as the primary biomarkers for progression. While observation is mandated for asymptomatic, non-progressive cases, the choice between buckling and vitrectomy for active detachments is often driven by surgeon preference rather than anatomical necessity. We propose an updated decision-making algorithm that integrates lens status, break localization, and vitreous findings to guide the surgical approach. Moving beyond a “one-size-fits-all” strategy, this review advocates for a personalized management plan that balances anatomical success with long-term quality of life.

## 1. Introduction

Acquired (senile) retinoschisis is a degenerative condition of the peripheral retina, historically described as a benign finding. In patients over age 40, the estimated prevalence ranges from 4% to 22%, with a higher incidence observed in the elderly population [1,2,3]. The condition is characterized by the splitting of the neurosensory retina, typically at the level of the outer plexiform layer, creating an inner and outer layer separated by a cavity filled with viscous mucopolysaccharides. The inferotemporal location is most common [1,2,3].

While the majority of cases remain stable and asymptomatic, a small subset of patients develops complications that threaten vision [1,2,3,4,5]. The most critical of these is the development of a retinoschisis-associated retinal detachment (R-RD). This specific entity must be distinguished from simple “schisis-detachment”, where fluid accumulates without full-thickness communication. True R-RD occurs only when breaks develop in both the inner and outer retinal layers, allowing liquefied vitreous to migrate into the subretinal space, potentially leading to rapid progression and macular involvement [3].

Historically, the management of R-RD has been controversial. In the pre-OCT era, distinguishing between a bullous schisis and a retinal detachment was often based on subtle clinical cues, leading to diagnostic uncertainty. Furthermore, the surgical landscape has shifted dramatically over the last decade. Scleral buckling (SB), once the undisputed gold standard, has seen a decline in usage, replaced by pars plana vitrectomy (PPV) due to the latter’s steeper learning curve and technological refinements. This review aims to critically analyze the current evidence comparing these techniques and proposes a modern therapeutic algorithm that balances anatomical success with functional quality of life.

## 2. Pathophysiology and Classification

### 2.1. Histopathology: Typical Versus Reticular Schisis

Acquired retinoschisis is fundamentally a senile degenerative process involving the peripheral retina. Histopathologically, it represents a coalescence of microcystoid degeneration, leading to the separation of retinal layers. While often grouped together clinically, two distinct histological subtypes exist, as originally classified by Straatsma and Foos [6]:

- Typical Cystoid Retinoschisis: The splitting occurs within the outer plexiform layer (OPL). It is characterized by a smooth, fusiform elevation of the inner layer. The schisis cavity is traversed by prominent pillar-like structures (Müller cell columns) that bridge the inner and outer layers. This form is usually confined to the pre-equatorial retina and rarely progresses to detachment.
- Reticular (Bullous) Retinoschisis: The splitting occurs more internally, at the level of the nerve fiber layer (NFL) or the ganglion cell layer. This form is clinically more significant as it tends to be bullous, extends posterior to the equator more frequently, and is more commonly associated with outer layer breaks.

In both forms, the inner layer (inner leaf) contains blood vessels and the internal limiting membrane (ILM), leading to the characteristic “white-with-pressure” or “snowflake” appearance due to the remnants of Müller cell footplates. The outer layer (outer leaf) is adjacent to the RPE and is the site where critical breaks occur.

### 2.2. The Biochemical Environment: Viscosity as a Barrier

A defining feature of retinoschisis, which distinguishes it from chronic retinal detachment, is the composition of the subretinal/intraschisis fluid. The cavity is filled with a viscous, mucopolysaccharide-rich fluid, primarily hyaluronic acid, which is produced by the decaying neuroretinal elements [7].

This high viscosity has two major clinical implications. First, it acts as a limiting factor for progression. Even in the presence of breaks, the fluid is too viscous to rapidly dissect the subretinal space, explaining the slow evolution of “schisis-detachments.” Secondly, the high viscosity represents a surgical challenge, especially during SB. In fact, external drainage is often complicated because the fluid does not flow freely through a sclerotomy, necessitating larger punctures or active aspiration [8].

### 2.3. Classification of Complications: The “Two-Hit” Mechanism

To guide surgical decision-making, it is imperative to distinguish between three clinical entities. Confusion often arises from inconsistent terminology in older literature. We adopt this classification refined by modern OCT findings [4,9,10]:

1. Isolated Retinoschisis: Splitting of layers without subretinal fluid accumulation outside the schisis cavity. Inner layer breaks (ILBs) are common (occurring in ~50–70% of cases) but are benign in isolation.
2. Schisis-Detachment (Non-Rhegmatogenous): Fluid from the schisis cavity accumulates in the subretinal space through small outer layer defects, but there is no communication with the vitreous cavity. The fluid remains viscous, and the detachment is typically immobile, convex, and demarcated by pigmented lines. This condition is often self-limiting.
3. Retinoschisis-Associated Retinal Detachment (R-RD): This is a progressive, vision-threatening, rare form, with an incidence of 0.05% in patients with degenerative RS [1]. It follows a “two-hit” mechanism: the formation of an outer layer break (OLB) (Hit 1), and the presence of an inner layer break (ILB) or extensive vitreous traction (Hit 2).

Crucially, the presence of both breaks creates a through-and-through channel. This allows liquefied vitreous (low viscosity) to bypass the schisis cavity and enter the subretinal space, converting a stable schisis into a rapidly progressive rhegmatogenous detachment.

The detection of OLBs is the single most important prognostic factor. Unlike inner layer breaks, which are small and circular, OLBs are often large, rolling, and posterior. Their large size is attributed to the lack of structural support in the thin outer necrotic layer and the tensile forces exerted by the elevated inner layer via Müller cell pillars.

## 3. Diagnostic Imaging: The Era of Widefield Tomography and AI

Historically, the diagnosis of retinoschisis relied on binocular indirect ophthalmoscopy combined with scleral depression. While classic signs—such as the “beaten metal” appearance, surface smoothness, and lack of shifting fluid—are well-described, clinical examination alone has low sensitivity for detecting small, peripheral OLBs. A recent comparative study demonstrated that up to 30% of OLBs identified on widefield imaging were missed during standard fundoscopy due to their anterior location or obstruction by the inner schisis layer [11]. Standard Spectral-Domain OCT (SD-OCT), limited by a 30–50° field of view, despite its usefulness, Refs. [9,12,13] often fails to capture the critical peripheral pathology where the schisis originates. While less common, B-scan ultrasonography remains useful in eyes with media opacities to distinguish the thin, mobile membrane of a detachment from the thicker, immobile membrane of a schisis [14].

In the era of multimodal imaging, the ultra-widefield autofluorescence and wide-field infrared (IR) imaging are quick, noncontact and non-invasive methods to compare RS, RD, and RS/RD [15,16,17]. On autofluorescence imaging, RS usually appears isoautofluorescent, whereas RD is often hypoautofluorescent [15,18]. The advantages of wide-field IR imaging are to capture the full extent of lesions and easily monitor their progression. Moreover, photographs can be acquired in poorly or nondilated pupils and do not require stable fixation of patients [16].

The introduction of ultra-widefield (UWF) imaging systems integrated with Swept-Source OCT (SS-OCT) has shifted the diagnostic paradigm from “pattern recognition” to “tomographic confirmation”. The longer wavelength of SS-OCT (~1050 nm) provides superior tissue penetration, allowing visualization of the vitreoretinal interface, outer retina and choroid even through the opaque, viscous fluid of a schisis cavity [17]. These findings can significantly influence clinical and therapeutical decisions, reducing unnecessary interventions [19,20,21].

Modern management is now guided by three specific tomographic biomarkers [9,10]:

- The “Bridging Columns” Sign:

UWF-OCT definitively identifies the pathognomonic splitting of retinal layers. The inner and outer layers are connected by stretched Müller cell columns (pillars). Visualization of these columns is important for distinguishing retinoschisis from chronic retinal detachment, where such bridging elements are absent

- Virtual Scleral Depression:

UWF-OCT allows for peripheral scanning that mimics scleral depression without patient discomfort. This “virtual depression” is critical for identifying Occult OLBs—small, full-thickness defects in the outer layer that are the primary drivers of R-RD progression.

- The “Schisis-RD” Transition Zone:

Widefield scans can visualize the transition zone where the smooth, immobile dome of the schisis evolves into the corrugated, mobile membrane of a rhegmatogenous detachment. This transition often correlates with the precise location of the outer layer break.

The updated differentiation criteria based on UWF-OCT are illustrated in Table 1.

The integration of artificial intelligence (AI) into widefield imaging represents the newest frontier in retinal diagnostics. Deep learning (DL) algorithms are showing promising results in automating the detection of peripheral retinal lesions that human graders might overlook.

A recent study has demonstrated that weakly supervised DL models could localize RD regions on UWF images with precision comparable to general ophthalmologists (89.1%), paving the way for automated screening of peripheral retinal degenerations [22]. A 2024 study developed a DL system achieving an area under the receiver-operating characteristic curve (AUC) of 0.975 for detecting retinal breaks and detachments on UWF images, demonstrating that AI can serve as a reliable “second reader” to prevent missed breaks [11]. Furthermore, in 2025, researchers introduced a PraNet-based machine learning model capable of pixel-level segmentation of retinal breaks in UWF images. This level of granular segmentation is crucial for R-RD, where the breaks are often multiple and clustered [23].

While these tools are currently used primarily for RD, their application to retinoschisis is imminent. AI-driven segmentation of the “outer retina” on OCT scans is expected to become a standard tool for identifying occult OLBs, potentially reducing the threshold for surgical intervention in “high-risk” asymptomatic patients.

Nevertheless, while the integration of artificial intelligence and ultra-widefield imaging represents a significant advancement in ophthalmic research, their routine use for degenerative retinoschisis is still in its early stages. Currently, these modalities are primarily confined to research settings rather than daily clinical practice. Further validation is required before these promising technological developments can transition into standardized clinical diagnostic protocols.

## 4. From Observation to Intervention: Defining the Surgical Threshold

The management of retinoschisis is rooted in the seminal long-term observations by Byer [1], who demonstrated that acquired retinoschisis is inherently benign in the majority of cases. In his cohort of 218 eyes followed for an average of nine years, zero cases of localized “schisis-detachment” progressed to clinical rhegmatogenous retinal detachment (R-RD). Therefore, the mere presence of subretinal fluid (SRF) is not an absolute indication for surgery. The presence of pigmented demarcation lines at the posterior border of the detachment is a robust biomarker of chronicity and a sign of stability, indicating that the RPE has had time to react to the static fluid. In these cases, even if the lesion is extensive, observation is the gold standard [1,4,24].

The transition from observation to surgery is primarily driven by documented anatomical progression, which should not be assessed solely by fundoscopy but also confirmed with multi-modal imaging.

Surgical intervention is indicated when monitoring reveals:

1. Posterior Extension: Fluid crossing the equator and extending toward the vascular arcades.
2. Breakthrough of Viscosity: A rapid change in the lesion’s morphology—from a smooth, convex dome (viscous fluid) to a corrugated, mobile membrane (liquefied vitreous). This morphological shift indicates that the “viscous barrier” has been breached by liquefied vitreous through an OLB, accelerating the rate of progression.
3. Collapse of Schisis Cavity: Paradoxically, the flattening of the inner schisis layer concurrent with an increase in subretinal fluid suggests that the intraschisis fluid has drained into the subretinal space, a precursor to rapid R-RD.

Regarding the visual acuity (VA), it is often a lagging indicator in R-RD, because if the macula is involved, surgery is mandatory, while in macula-ON cases, VA is typically preserved and the decision to operate is prophylactic to prevent foveal involvement [25,26].

Acquired retinoschisis causes an absolute scotoma. Symptomatic visual field (VF) loss is a more sensitive indicator of progression than VA. A supero-nasal field defect (corresponding to the common infero-temporal schisis) that enlarges toward the central 10–20 degrees is a strong indication for surgery.

Moreover, the onset of new photopsia (flashes) or an increase in floaters suggests acute vitreous traction or the formation of new retinal breaks, warranting immediate surgical consideration regardless of the previous stability of the schisis (Table 2).

A common misconception is that the use of a “barrier laser” is to delimit a progressive schisis. The literature consistently advises against this practice. Retinopexy applied to the bed of a retinoschisis can induce iatrogenic outer layer breaks due to tissue necrosis or thermal contraction, potentially converting a stable schisis into a fulminant R-RD [27]. Laser is only indicated in very specific cases to surround a frank retinal detachment [28], but generally, if the lesion is dangerous enough to laser, it is often dangerous enough to require vitrectomy or buckling.

## 5. Surgical Management: Techniques, Outcomes, and Current Trends

The surgical management of retinoschisis-associated retinal detachment (R-RD) has evolved into a dichotomy between the classical “ab externo” approach (SB, scleral buckling) and the modern “ab interno” approach (PPV, pars plana vitrectomy).

For phakic eyes with anterior OLBs, SB is the preferred intervention to preclude cataractogenesis and preserve accommodation. This node is further supported by Grigoropoulos et al. [29], who reported an 86% single-surgery success rate for anterior breaks managed with SB, and Avitabile et al. [30], who demonstrated that carefully placed buckling elements combined with external fluid drainage yield exceptional anatomical outcomes (92% success rate) even for selected posterior breaks.

In 2014, Gotzaridis et al. compared the success rate of SB with PPV in a cohort of 30 patients with RS [31]. The authors highlighted the importance of tailoring the approach based on the vitreous status, favoring SB in the absence of a posterior vitreous detachment (PVD) to avoid inducing complex vitreous traction, while reserving pars plana vitrectomy (PPV) for eyes with an established PVD. Gotzaridis et al. found a significant difference in the primary success rate between both groups, with the SB group being associated with better results, although, there was no difference in the final reattachment rate [31].

While historical data favored SB, recent surveys indicate a significant shift: currently, PPV is the most frequently used, especially for cases of OLB located posterior to the equator [28,32,33,34].

Garneau et al. [32] compared the anatomical success rate of first surgery between PPV alone and combination PPV-SB in R-RD. Standard PPV and combined PPV-SB showed similar surgical outcomes in repairing the R-RD. Moreover, they showed that the anatomical success rate of primary surgery for R-RD was significantly lower compared to rhegmatogenous retinal detachment (RRD). The primary success rate of surgery could also be worse in R-RD patients because of the surgery difficulty itself as retinal breaks and holes are more easily missed during procedure and can eventually be responsible for retinal re-detachments [30,31,32].

In scenarios of high complexity—such as multiple/bilayer breaks, PVR, vitreous hemorrhage, or extreme peripheral pathology with difficult visualization—a PPV or a combined PPV/SB approach is necessary. Stem et al. [33] demonstrated that for complex bilayer breaks, combining PPV with an encircling buckle offers superior primary outcomes compared to PPV alone. Recent large-scale surveys and comparative cohorts in the modern era, including Garneau et al. [32] and Liao et al. [34], confirm that R-RD poses significant surgical challenges with generally lower primary success rates than standard detachments; nevertheless, strictly adhering to an anatomical stratification ensures that both PPV and combined PPV/SB remain the most robust primary repair methods for these complex presentations.

In the context of management strategies, a brief discussion on pneumatic retinopexy (PR) is relevant. Some authors have reported PR as an effective, minimally invasive option for the treatment of R-RD [35,36]. However, the authors emphasize that PR’s long-term success critically depends on the meticulous preoperative identification and rigorous sealing of all retinal breaks. Failure to detect occult linear tears, particularly in the inner retinal layer, can lead to early re-detachment and treatment failure, ultimately requiring secondary PPV.

This section critically compares SB and PPV techniques in the context of modern microsurgery.

### 5.1. Scleral Buckling (SB): The “Physiologic” Approach

Despite the decline in usage, SB remains the theoretical gold standard for phakic patients, particularly those with localized, inferior pathology.

SB treats the pathology by bringing the RPE into contact with the neurosensory retina and the OLBs from the outside, relieving vitreous traction without invading the eye or accelerating cataract formation.

The most significant technical challenge in R-RD is the high viscosity of the intraschisis fluid. Unlike the low-viscosity fluid of a standard RRD, schisis fluid often fails to drain through a standard sclerotomy. Experienced surgeons now advocate for active external drainage using a larger needle (26G) or multiple puncture sites under direct visualization. Alternatively, some proponents of the “non-drainage technique” suggest that if the buckle height is sufficient to close the OLB, the RPE pump will eventually reabsorb the fluid, although this process is significantly slower than in standard RD [30].

SB is the preferred choice for young, phakic patients with anterior or equatorial OLBs where preserving accommodation is paramount [4,29,31,32,33,34,35,36,37].

When an OLBs cannot be localized during a primary scleral buckling procedure, surgeons are faced with a significant intraoperative challenge. In such scenarios, if an external approach is maintained, the traditional strategy involves the placement of a broad segmental buckle or an encircling band designed to indent and support the entire bed of the schisis cavity, particularly targeting its posterior margin where occult breaks frequently reside. This is typically combined with external drainage of subretinal fluid and broad cryopexy. However, contemporary vitreoretinal practice increasingly favors intraoperative conversion to PPV. An internal approach, utilizing wide-field viewing systems and endoillumination, provides superior visualization of the retinal architecture, thereby maximizing the probability of identifying occult outer layer defects. Furthermore, PPV allows for the controlled internal drainage of schisis and subretinal fluid, followed by targeted endolaser photocoagulation and endotamponade, optimizing anatomical success rates.

### 5.2. Pars Plana Vitrectomy (PPV): The “Controlled” Approach

PPV appears to be the procedure of choice for extensive R-RD with a large OLB or when extending posterior to the equator [31,32]. The potential benefits of PPV in such cases are that it allows good visualization and access to the break edges and enables both controlled internal drainage of subretinal fluid and endolaser [30,31,32,33,34,35,36,37,38].

PPV has become the dominant technique due to the widespread adoption of small-gauge (25G/27G) systems and wide-angle viewing systems.

A critical step in R-RD vitrectomy is the identification of all OLBs. The use of triamcinolone acetonide is strongly recommended not only to ensure a complete posterior vitreous detachment (PVD) induction but to visualize vitreous schisis cavities.

A subject of debate is how to handle the inner schisis layer.

- Inner Leaf Retinectomy: In cases of bullous schisis obscuring the view or preventing tamponade fill, a retinectomy of the inner layer is performed. This converts the complex dual-layer detachment into a simple single-layer detachment, facilitating internal drainage and laser photocoagulation to the OLBs [39]. In juvenile retinoschisis it was found that vitreous surgery and removal of the inner wall of the schisis cavity might help to reduce that progression and to achieve and maintain better central visual acuities [40]. The unroofing of the schisis cavity during PPV ensures relief of residual vitreous tractions and has no consequences on peripheral visual field, because of the complete scotoma in RS area.
- Conservation: Most surgeons leave the inner layer intact if it does not exert traction. Some authors reported the development of proliferative vitreoretinopathy (PVR) detachment and macular pucker after inner layer retinectomy. They speculated on the role of inner layer resection on inducing glial proliferation, which may not only be related to the retinectomy but also to the increased access of retinal pigment epithelial cells to the vitreous cavity, changing the schisis detachment to a true RRD [28].

PPV allows for active internal drainage of the viscous fluid through the OLB or a retinotomy. This provides immediate reattachment, which is gratifying for the surgeon and allows for precise intraoperative laser.

Gas (SF6 or C3F8) is usually sufficient, and silicone oil is reserved for cases with PVR or inability to posture.

### 5.3. Complication Profile and Quality of Life

- The “Buckle Complications”: SB is associated with refractive changes (myopic shift of ~1.00 D–2.00 D), diplopia (extraocular muscle restriction), and long-term risk of hardware extrusion or infection.
- The “Vitrectomy Cascade”: In phakic eyes, PPV almost invariably leads to nuclear sclerotic cataract progression. For a 40-year-old patient, the loss of accommodation (presbyopia) following cataract surgery is a significant quality of life reduction that must be weighed against the surgical success.
- Iatrogenic Breaks: PPV carries a risk of iatrogenic retinal breaks (3–4%) during the induction of PVD, especially in adhesive myopic eyes, which can complicate the course.

### 5.4. Combined Surgery (SB + PPV)

The combined approach is reserved for the most complex scenarios [33]. It is indicated in:

1. Multiple OLBs spanning different quadrants (where a single buckle is insufficient).
2. Giant retinal tears associated with schisis.
3. Chronic R-RD with PVR: Where internal peeling and external support are both required to counteract retinal shortening.

## 6. Discussion: Towards a Personalized Surgical Algorithm

The management of R-RD represents a microcosm of the broader evolution in vitreoretinal surgery: the tension between classical “ab externo” techniques and modern “ab interno” technologies.

Our review of the current literature reveals a paradox: while PPV has become the dominant surgical approach globally—driven by the versatility of small-gauge instrumentation and the “comfort zone” of younger surgeons—comparative data do not support its superiority in terms of primary anatomical success [28,31].

The most critical finding from recent comparative series is that the choice of surgery impacts the patient’s refractive status more than the retinal status.

- The Phakic Imperative: In young, phakic patients, the “vitrectomy cascade” (cataract formation, loss of accommodation, potential YAG complications) represents a significant morbidity. For these patients, SB remains the biologically superior choice. It addresses the pathology by supporting the outer layer breaks without disturbing the lens-iris diaphragm or the vitreous body. The decline in SB training in fellowship programs is a concern that risks depriving phakic patients of this optimal treatment option.
- The Pseudophakic Pragmatism: Conversely, in pseudophakic eyes, the rationale for SB diminishes. PPV offers a controlled environment, superior visualization of posterior breaks, and avoids the refractive unpredictability (astigmatism/myopic shift) associated with buckling hardware.
- Posterior OLBs: Regardless of lens status, posterior breaks are difficult to buckle effectively, and PPV is preferred here to ensure adequate tamponade.

To establish a robust, evidence-based foundation for the proposed clinical algorithm, it is essential to contextualize the surgical decision-making process within the existing literature. Given the relative rarity of progressive retinoschisis-associated retinal detachment (R-RD), the available surgical evidence predominantly consists of retrospective cohorts and case series with limited sample sizes, and there are currently no randomized clinical trials to provide absolute treatment guidelines. Consequently, we carefully selected a representative panel of key comparative and surgical outcome studies that specifically address the critical decision nodes outlined in our management pathway.

The selected literature was chosen to reflect the evolution and stratification of surgical techniques: it spans from foundational reports validating the efficacy of SB for localized, anterior breaks, to contemporary comparative analyses evaluating PPV and combined approaches (PPV/SB) for highly complex or pseudophakic presentations. This purposeful selection aims to provide a clear, evidence-based rationale for how specific anatomical variables—namely, the topography of OLBs, lens status, and the presence of PVR—directly dictate the optimal surgical modality in our flowchart. Table 3 summarizes these pivotal studies and explicitly links their key findings to the stratification logic of the proposed algorithm.

## 7. A New Algorithm

**Figure 1:**
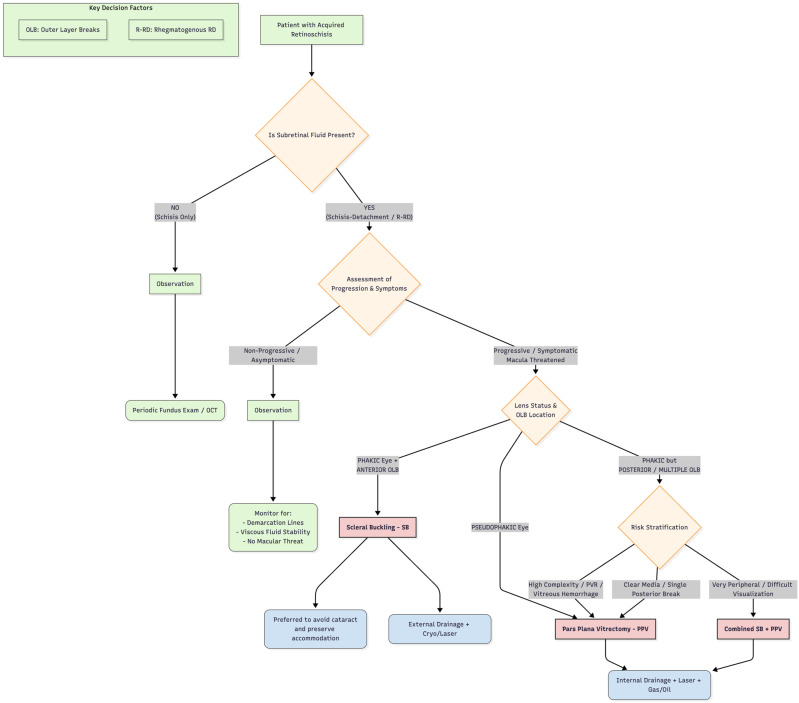
Algorithm based on current literature. Note that OLBs are the primary pathogenetic mechanism for R-RD. If OLBs cannot be identified pre-operatively despite wide-field imaging and indentation, PPV is often preferred to allow intraoperative identification. Note that if the patient already exhibits lens opacities, the surgical preference shifts from SB to PPV. The combined surgery (SB + PPV) is reserved for complex cases with PVR or inferior breaks in phakic eyes where tamponade support is challenging. Legend: Outer layer breaks (OLBs); retinoschisis-retinal detachment (R-RD); pars plana vitrectomy (PPV); scleral buckle (SB); proliferative vitreoretinopathy (PVR).

The primary objective of this review is to formulate a clinical decision-making algorithm to assist surgeons in the management of this complex pathology (Figure 1). The proposed algorithm is grounded in the current cited literature, as well as the collective clinical expertise of the authors. A comprehensive summary of these key surgical outcome studies supporting our decision nodes is provided in Table 3.

The clinical management of acquired retinoschisis dictates a highly stratified, stepwise approach predicated on the presence of subretinal fluid, disease progression, and patient-specific anatomical variables. The primary decision node hinges on the detection of subretinal fluid; isolated retinoschisis mandates conservative management via continuous observation, utilizing periodic fundus examinations and OCT. If a schisis-detachment or R-RD is confirmed, the therapeutic pathway bifurcates based on clinical progression and symptomatology. Asymptomatic, non-progressive cases without macular threat are safely monitored for spontaneous stabilization, evidenced by demarcation lines and viscous fluid stability. Conversely, progressive or symptomatic detachments threatening the macula necessitate surgical intervention. The selection of the surgical modality is subsequently dictated by lens status and the topography of OLBs.

SB, often paired with external drainage and cryotherapy, is the preferred intervention for phakic eyes with anterior OLBs, primarily to preclude cataractogenesis and preserve accommodation [30,31].

However, a crucial clinical caveat must be addressed regarding phakic eyes: if the patient is within the cataractogenous age group or already exhibits early lens opacities, the surgical preference shifts from scleral buckling to pars plana vitrectomy (PPV). Performing a PPV in these cases—often combined with phacoemulsification and intraocular lens implantation—not only avoids the technical difficulties of a subsequent cataract surgery in a vitrectomized and buckled eye, but also guarantees optimal intraoperative visualization of the peripheral retina to identify occult outer layer breaks.

In pseudophakic eyes, or phakic eyes presenting with complex features—such as posterior or multiple OLBs, PVR, or vitreous hemorrhage—a PPV or a combined PPV/SB approach is frequently preferred to ensure adequate peripheral base support and relieve vitreous traction [31,32,33,34].

Ultimately, in instances of extreme peripheral pathology where visualization is compromised, a combined SB and PPV approach is indicated. All vitrectomy-based pathways converge on a standardized resolution involving internal fluid drainage, laser application, and endotamponade utilizing either gas or silicone oil to ensure long-term retinal apposition.

## 8. Conclusions

Acquired retinoschisis with associated detachment is a unique clinical entity that demands high-level diagnostic precision using OCT. The transition from observation to surgery should be reserved for progressive, symptomatic cases with confirmed OLBs. While the trend towards vitrectomy is undeniable, SB remains an indispensable tool in the retinal surgeon’s armamentarium, particularly for young phakic patients. We propose that the modern vitreoretinal surgeons prefer a tailored strategy based on lens status and break localization, as outlined in our algorithm, compared to a “one-size-fits-all” vitrectomy approach. We advocate for a hybrid approach where surgeon expertise in both techniques allow for personalized patient care.

## 9. Method of Literature Search

A comprehensive and systematic literature search was conducted across two major electronic databases, PubMed and Embase, to identify relevant studies published between January 1958 and March 2026. The search strategy was designed using a combination of Medical Subject Headings (MeSH) and free-text keywords to maximize sensitivity. The Boolean search string included: (“retinoschisis” OR “degenerative retinoschisis” OR “senile retinoschisis”) AND (“management” OR “surgery” OR “imaging” OR “multimodal imaging” OR “retinal detachment”).

To ensure the clinical relevance and quality of the review, specific eligibility criteria were established prior to the screening process. Inclusion criteria were defined as follows: (1) peer-reviewed articles reporting on the diagnosis, multimodal imaging features, clinical course, or surgical management of degenerative retinoschisis and retinoschisis-associated retinal detachment; (2) study designs including prospective and retrospective observational studies, randomized controlled trials, case series, and previous comprehensive reviews; and (3) articles published in the English language. Exclusion criteria consisted of: (1) purely experimental animal or in vitro studies; and (2) conference abstracts, editorials, or letters to the editor lacking primary clinical data.

The screening process was conducted in two independent phases. Initially, two independent reviewers screened the titles and abstracts of all retrieved articles to remove duplicates and broadly irrelevant publications. In the second phase, the full-text versions of the remaining potentially eligible articles were retrieved and thoroughly evaluated against the predefined inclusion and exclusion criteria. Any discrepancies between the reviewers regarding article eligibility were resolved through discussion and consensus.

## Figures and Tables

**Table 1:** UWF-OCT based criteria to differentiate retinoschisis (RS) and chronic rhegmatogenous retinal detachment (RD).

Feature	Retinoschisis	Chronic Rhegmatogenous RD
Retinal Thickness	Thickened (split layers visible)	Normal or thinned (atrophic)
Bridging Columns	Present (Pathognomonic)	Absent
Intra-retinal Cysts	Common in the inner wall	Cystoid macular edema (if macula-on)
Mobility	Immobile/minimal shifting	Mobile/shifting fluid
Pigment	Rare in the cavity	Demarcation lines common at border

**Table 2:** Absolute indications for surgical intervention in retinoschisis-retinal detachment.

Indication Category	Specific Criteria
Anatomical	Confirmed R-RD (inner + outer layer breaks). Progressive extension of fluid beyond the vascular arcades. Absence of demarcation lines in a progressing lesion.
Functional	Detachment of the macula (Macula-OFF). “Macula-Threatened” status (fluid within 1–2 disc diameters of fovea). Symptomatic visual field loss encroaching on central fixation.
Complications	Vitreous hemorrhage (from bridging vessels). Associated PVR (proliferative vitreoretinopathy).

**Table 3:** Summary of key surgical outcome studies for retinoschisis-associated retinal detachment (R-RD).

Study (Author, Year)	Cohort & Study Design	Surgical Modalities Evaluated	Key Outcomes/Single Surgery Success (SSAS)	Relevance for the Proposed Algorithm
Grigoropoulos et al., 2006 [29]	30 eyes; progressive R-RD stratified by OLB location and PVR.	SB, PPV or combined.	SSAS: 85% (SB for anterior OLB); 67% (PPV for posterior OLB); 0% (PPV for PVR > Grade B).	Strongly supports the central node of the algorithm: stratifying surgical choice directly based on OLB topography and complexity.
Avitabile et al., 2010 [30]	37 eyes; progressive symptomatic R-RD with OLB at posterior to equator.	SB with external drainage of subretinal and schisis-cavity fluid.	SSAS: 92%.	Demonstrates that tailored SB with external fluid drainage is a highly effective pathway, even for selected posterior breaks.
Gotzaridis et al., 2014 [31]	30 eyes; symptomatic degenerative R-RD, stratified by posterior vitreous detachment (PVD).	SB (no PVD) vs. PPV (with PVD) vs. combined.	Overall SSAS: 70%. Grp I (SB): 76%. Grp II (PPV): 62%.	Validates the decision to tailor surgery: SB is favored when PVD is absent, to avoid inducing complex vitreous traction.
Stem et al., 2019 [33]	37 eyes; complex R-RD with both inner and outer layer breaks.	SB, PPV, or combined PPV/SB.	Overall SSAS: 65%. SB: 71%. PPV: 44%. PPV/SB: 71%.	Directly justifies the “High Complexity” node: in complex bilayer breaks, combined PPV/SB or standalone SB offers superior primary outcomes compared to PPV alone.
Garneau et al., 2022 [32]	41 R-RD eyes vs. 1661 standard RD eyes.	Primary PPV vs. combined PPV/SB.	SSAS was comparable between PPV (79%) and PPV/SB (80%) for complex R-RD cases and RRD. R-RD was associated with worse final VA.	Confirms that PPV and PPV/SB are valid primary repair methods for complex/pseudophakic cases.
Liao et al., 2022 [34]	16 eyes; 17-year retrospective survey from a large academic center.	SB (10 eyes), PPV (3 eyes), combined PPV/SB (3 eyes).	SSAS: 56.2%. Final anatomical success: 100%.	Highlights the surgical challenge of R-RD; reinforces that strict algorithmic stratification is necessary to achieve ultimate anatomical success.

Abbreviations: PPV = pars plana vitrectomy; PVD = posterior vitreous detachment; R-RD = retinoschisis-associated retinal detachment; OLBs = outer layer breaks; PVR = proliferative vitreoretinopathy; RD = retinal detachment; SB = scleral buckle.

## Data Availability

The original contributions presented in this study are included in the article. Further inquiries can be directed to the corresponding author.
